# 

**Published:** 1875-10

**Authors:** 


### Books and Pamphlets Received.

The Cholera Epedemic of 1873, in the United States. Reports. Prepared the Direction of the Surgeon General. By Ely McClellan, M. D., John C. Peters, M. D., John S. Billings, M. D., and John M. Woodworth, M. D. Washington, Government Printing Office, 1875.

Annual Report of the Supervising Surgeon of the Marine Hospital Service of the United States, for the Fiscal Year 1874. Washington, Government Printing Office, 1874.

A Treatise on Human Physiology; Designed for the Use of Students and Practitioners of Medicine. By John C. Dalton, M. D. Sixth Edition, Revised and Enlarged. Philadelphia: Henry C. Lea, 1875 Buffalo: T. Butler & Son.

On Poisons in Relation to Medical Jurisprudence and Medicine. By Alfred Swaine Taylor, M. D., F. R. S. Third American from the Third English Edition. Philadelphia: Henry C. Lea, 1875. Buffalo: T. Butler & Son.

Lectures on Syphilis and on some Forms of Local Disease, Affecting Principally the Organs of Generation. Henry Lee, Professor of Surgery at the Royal College of Surgeons of England. Philadelphia: Henry C. Lea, 1875. Buffalo: T. Butler & Son.

A Manual of Minor Surgery and Bandaging. By Christopher Heath, F. R. C. S. Fifth Edition. Philadelphia: Lindsay & Blakiston, 1875. Buffalo: T. Butler & Son.

The Physicians Visiting List for 1876. Philadelphia: Lindsay & Blakiston.

The Mucous Membrane of the Uterus, with Special Reference to the Development and Structure of the Deciduæ. By Geo. J. Engelman, A. M., M. D. New York: Wm. Wood & Co., 1875. Buffalo: H. H. Otis.

Clinical Lectures and Essays. By Sir James Paget, Bart. Edited by Howard Marsh, F. R. C. S. New York: D. Appleton & Co., 1875. Buffalo: Herger & Ulbrich.

On Paralysis from Brain Disease in its Common Forms. By H. Charlton Bastian, M. A., M. D. New York: D. Appelton & Co., 1875. Buffalo: Herger & Ulbrich.

A Report on a Plan for Transporting Wounded Soldiers by Railway in Time of War. By George A. Otis, Asst. Surg., U. S. Army, Washington; Surgeon General's Office, 1875.

Transactions of the Medical Society of the District of Columbia for July and October, 1875.

### Contents of October Number.

### ORIGINAL.

THE BUFFALO

### Medical and Surgical Journal

PUBLISHED MONTHLY,

**Containing Original Articles, Reports of Medical Societies and Hospitals, Editorial, Reviews, Correspondence, Army News, etc., etc; including the usual variety of Medical Periodical Publications. Specimen copies sent on application.**

Address Buffalo Medical & Surgical Journal, Buffalo, N. Y.

TERMS OF SUBSCRIPTION, - $3.00 PER YEAR, IN ADVANCE.

